# Pathological and Functional Amyloid Fibrils—Part I

**DOI:** 10.3390/ijms23126447

**Published:** 2022-06-09

**Authors:** Irina M. Kuznetsova, Konstantin K. Turoverov

**Affiliations:** Laboratory of Structural Dynamics, Stability, and Folding of Proteins, Institute of Cytology, Russian Academy of Sciences, 4 Tikhoretsky Ave., 194064 St. Petersburg, Russia; kkt@incras.ru

Amyloid fibrils have been known to researchers for a long time. The first mention of amyloid fibrils, which can be found in PubMed, dates back to 1861 [1]. But only at the beginning of the new century did the number of works devoted to the study of amyloid fibrils begin to grow significantly. One explanation for this growth seems to be that increased life expectancy has made the problem of senile neurodegenerative diseases in developed countries comparable to cardiovascular disease and carcinogenesis, and significant funds have been devoted to the study of amyloid fibrils and related diseases. In the last 20 years researchers have made great progress in amyloid fibrils investigation.

If at first it seemed that all fibrils are the same and are regular, β-sheet-enriched, long nanosized protein aggregates with β-strands running perpendicular to the long axis of the fibril, then by now it has become obvious that amyloid fibrils formed by different amyloidogenic proteins are significantly different in structure, rate of formation, tendency to form larger aggregates, toxicity, etc. Furthermore, it turned out that not all amyloid fibrils are harmful. Many amyloid fibrils are non-toxic and have important functions [2].

The more we learn about amyloid fibrils, the more the horizon for future research expands. The number of publications on this topic is not decreasing but steadily growing.

The aim of this Special Issue was to collect, under one cover, the most important modern ideas about the mechanism of formation, structure, stability, prone-to-plaque formation, and other important features of a wide range of amyloid fibrils. The collection includes only 5 articles, but it highlights a number of burning problems, each of which certainly adds new touches to the “portrait of amyloid fibrils”.

The collection is opened by the paper by Lin et al. in which the cross-species seeding of amyloid A (AA) amyloidosis was studied [3]. This amyloidosis, one of the most common forms of clinically important amyloidosis, is related to chronic inflammatory diseases and chronic infections. Serum amyloid A (SAA) plays a vital step in AA amyloidosis, being the most important precursor activated by the amyloid enhancing factor (AEF), which serves as a seed for fibril formation. In this study, the aggregation of mSAA mixed with AEFs from five animals (camel, cat, cattle, goat, and mouse) was visualized using QD nanoprobes. The results showed that AEFs shortened and promoted mSAA aggregation. A detailed comparison of amino acid sequences suggested that it was important to mSAA aggregation-promoting activity that the 48th amino acid was a basic residue (Lys) and the 125th amino acid was an acidic residue (Asp or Glu). These data imply that AA amyloidosis exhibits higher transmission activity among animals carrying a genetically homologous SAA gene. This information may provide a better understanding of amyloid disease and lead to the development of novel therapies [3].

One of the ways to prevent amyloidosis is the inhibition and the stop/slowdown of the growth of amyloid fibrils. For many amyloid fibrils formed by various proteins, amyloidogenic sequences, which are the seeds for aggregation and subsequent growth of amyloid fibrils, have been identified. However, it remains unclear why exactly these sequences are prone to aggregation and under what conditions this occurs. Effective action on amyloidogenic sequences can be considered as a way to stop amyloidosis, but for this it is necessary to know the mechanism of their action on these amyloidogenic sequences. Muvva et al., using force-field, semi-empirical, and density functional theory calculations, provided the theoretical basis of amyloid forming tendency of peptide sequences from amyloid beta and tau proteins [4].

Galkin and Sysoev have focused their review on the causes of amyloid fibrils formation [5]. They drew attention to the fact that only 5–10% of amyloidosis is determined by amyloidogenic mutations or the transfer of infectious amyloids (prions) between organisms. Whereas the most common group of so-called sporadic amyloidoses is associated with abnormal aggregation of wild-type proteins. Some sporadic amyloidosis is induced against the background of certain pathologies, but in some cases the cause of amyloidosis is unclear. The authors present facts and hypotheses about the association of sporadic amyloidosis with vascular pathologies, trauma, oxidative stress, cancer, metabolic diseases, chronic infections, and COVID-19. Summarizing current data, the authors show that all sporadic amyloidosis can be considered as a secondary event that occurs against the background of diseases that provoke a cellular stress response. Various factors that cause a stress response provoke hyperproduction of the protein, a local increase in its concentration or modification, which contributes to amyloidogenesis. Thus, progress in the treatment of vascular, metabolic, and infectious diseases, as well as oncological diseases, should lead to a significant reduction in the risk of sporadic amyloidosis [5].

To the extremely interesting observations and generalizations of the authors, we would like to add that the studies reviewed by the authors do not explain how the local increase in protein concentration which is necessary for the initiation of amyloidogenesis occurs. At the same time, it is now known that the local concentration of proteins can occur in membraneless organelles, dynamic coacervates, which also emerge in response to stress as a result of liquid-liquid phase separation [6]. The coverage of the role of non-membrane organelles in the formation of amyloid fibrils would be a logical continuation of the review by Galkin and Sysoev.

Bobylev et al. considered one of the possible mechanisms of cytotoxicity of amyloid fibrils [7]. The authors studied the effect of amyloid aggregates of smooth muscle titin on cultures of smooth muscle cells. Aggregates have been shown to disrupt cell adhesion, which is accompanied by disorganization of the actin cytoskeleton and the formation of filopodia, lamellipodia, and stress fibers. Cells died after 72 h of contact with amyloid aggregates. The results of this work are important for understanding the mechanisms of cytotoxicity of amyloid fibrils [7].

Finally, the special issue is completed by an article by Stepanenko et al., which discusses the resistance of amyloid fibrils to proteolysis [8]. The authors studied trypsin-induced degradation of amyloid fibrils and showed that the mechanism of this process is qualitatively the same for all studied amyloids; however, the rate of fibril degradation depends on the structure of the amyloid-forming protein, as well as on the morphology and clustering of amyloid fibrils. The authors showed that the cytotoxicity of trypsin-treated amyloids not only does not decrease but may even increase, as for example for beta-2-microglobulin fibrils, which is not surprising in general, since it is known that protofibrils are often more cytotoxic compared to mature fibrils and, in addition, fibril fragments can be the embryos of new fibrils.
